# Cannabis, Weight, and Weight-Related Behaviors

**DOI:** 10.1007/s13679-025-00633-z

**Published:** 2025-05-08

**Authors:** Kasey P. S. Goodpaster

**Affiliations:** Cleveland Clinic Bariatric & Metabolic Institute, Cleveland Clinic Lerner College of Medicine of Case Western Reserve University, 9500 Euclid Ave./M61, Cleveland, OH 44195 USA

**Keywords:** Cannabis, Appetite, Substance use, Eating disorders, Binge eating, Physical activity, Weight, Obesity

## Abstract

**Purpose of Review:**

Review recent research regarding the relationship between cannabis use, weight, eating behaviors, eating disorders, and physical activity.

**Recent Findings:**

Cannabis, particularly the cannabinoid Δ9-tegrahydrocannabinol (THC), is associated with increased appetite, food cravings, overconsumption, and decreased physical activity. Cannabidiol (CBD) appears to be associated with decreased appetite. While cannabis use is not correlated with binge eating, cannabis use disorder is associated with loss of control eating. Despite cannabis’ association with unhealthy eating and sedentary behavior, most studies suggest that cannabis use is not associated with weight gain, or may even facilitate weight loss.

**Summary:**

The state of the literature regarding the relationship between cannabis, weight, and weight-related behaviors is complex. Most studies do not differentiate between cannabinoid profiles, routes of administration, or whether cannabis use is problematic. Patients presenting for weight management should be cautioned about cannabis, particularly THC, potentially increasing risk of unhealthy eating and activity patterns.

## Introduction

Both cannabis use and obesity have been increasing in recent years, with the expectation that both will continue to rise. The prevalence of obesity has doubled world-wide since 1990, and over 40% of adults in the United States (US) are now affected by obesity [1, 2]. Cannabis is the most commonly used drug globally [3]. According to a recent systematic review and metanalysis, there has been disproportionate increase in cannabis use world-wide, and relatively greater prevalence in countries where recreational and/or medical cannabis has been legalized [4]. Of note, research highlighting increased prevalence should be interpreted with the caveat that participants may be more willing to report cannabis use over time due to increased legalization. Over a third of US adults (36.3%) now report using cannabis over past month [5], as compared to 1.4% to 38.12% across 15 countries in North and South America, and 0.42% to 43.90% across 33 European countries [4]. In the US, the majority of states have legalized medical and/or recreational cannabis, and consistent with global trends, with increased legalization has come increased perceived acceptability, ease of access, and frequency of use [6]. In fact, the prevalence of daily or near daily use doubled between 2009 and 2018 to 4.7% of US adults [3].

Theoretically, cannabis use would increase body weight by way of influencing eating behaviors (what is colloquially referred to as “the munchies”) and reducing motivation for physical activity (particularly when strains known for their calming effects are used). However, there have been conflicting findings in the research. In this review, we summarize the state of the literature regarding the relationship between cannabis, eating behaviors, eating disorders, physical activity, and weight, respectively. We conclude with clinical recommendations and directions for future research.

Of note, cannabis use has many implications for patients before and after metabolic and bariatric surgery. This topic is beyond the scope of the present review and is covered in a recent paper endorsed by the American Society for Metabolic and Bariatric Surgery [7].

## Cannabis Overview

Cannabis products are derived from the plant species *Cannabis sativa*, *Cannabis indica*, and *Cannabis ruderalis.* Two primary compounds of cannabis include Δ9-tegrahydrocannabinol (THC) and cannabidiol (CBD). THC is primarily responsible for psychoactive effects including feelings of euphoria, increased appetite, cognitive impairment, and acute intoxication, whereas CBD is non-psychoactive and primarily responsible for therapeutic effects such as reduced pain and inflammation [8, 9].

THC activates endocannabinoid receptors CB1, which results in increased appetite and a heightened sensitivity to the hedonic properties of highly palatable food [10, 11], and to a lesser degree, CB2, which results in an anti-inflammatory response [12]. By contrast, CBD is an antagonist of CB1 and thus inhibits its hedonic effects [9], has been found to have anti-inflammatory and anti-oxidant effects, and appears to reduce appetite particularly in individuals with higher body max index (BMI) [8, 13].

Cannabis products vary in terms of the ratio of THC to CBD, which helps to explain mixed research findings regarding the relationship between cannabis, eating behaviors, and weight. Additionally, the subjective experience of cannabis appears to be dependent on the route of administration. For example, cannabis can be inhaled via smoking or vaping; ingested in the form of various foods/“edibles,” drinks, pills, lozenges, and tinctures; and topically applied in lotions or balms [14]. Inhaled routes of THC administration tend to lead to a rapid onset but shorter duration of increased appetite, whereas ingested routes lead to delayed and stronger increases in appetite that may last for hours. While research comparing routes of administration is limited, one recent laboratory study of 20 cannabis users found that oral THC consumption leads to higher levels of the hunger hormone, ghrelin, than smoked or vaped forms [15].

Other factors that appear to determine to what extent cannabis influences appetite, eating behaviors, and weight are frequency and duration of use. Long-term, frequent cannabis users often develop a tolerance to the acute effects of the substance, including appetite stimulation, that are more noticeable to those who have not used cannabis for a prolonged period of time [16]. Pharmacodynamic research suggests that chronic THC use leads to downregulation and desensitization of CB1, which helps to explain why appetite stimulation is suppressed in chronic users [16]. However, the frequency, dose, and duration of cannabis use needed to develop physiological tolerance is not yet known [16].

While long-term users may not experience significant appetite-stimulating effects, frequent, problematic cannabis use is of clinical relevance considering its relationship to eating disorders. Cannabis use disorder (CUD) is defined as a problematic pattern of cannabis use with clinically significant distress or impairment [17]. To meet diagnostic criteria, an individual must have at least two of 11 specified symptoms (e.g., persistent desires to cut down, strong cravings, tolerance, withdrawal, etc.) within a 12-month period (see Table 1) [17]. Globally, approximately 10% of those who use cannabis meet criteria for CUD [3]. As will be discussed later in this review, CUD appears to be more strongly related to eating disorders than does cannabis use that is not frequent or problematic.

**Table 1:** DSM-5-TR criteria for Cannabis use disorder [17]

**Criterion:** A problematic pattern of cannabis use leading to clinically significant impairment or distress, as manifested by at least 2 of the following, occurring within a 12-month period:
1. Cannabis is often taken in larger amounts or over a longer period than was intended 2. There is a persistent desire or unsuccessful efforts to cut down or control cannabis use 3. A great deal of time is spent in activities necessary to obtain cannabis, use cannabis, or recover from its effects 4. Craving, or a strong desire or urge to use cannabis 5. Recurrent cannabis use resulting in a failure to fulfill major role obligations at work, school, or home 6. Continued cannabis use despite having persistent or recurrent social or interpersonal problems caused or exacerbated by the effects of cannabis 7. Important social, occupational, or recreational activities are given up or reduced because of cannabis use 8. Recurrent cannabis use in situations in which it is physically hazardous 9. Cannabis use is continued despite knowledge of having a persistent or recurrent physical or psychological problem that is likely to have been caused or exacerbated by cannabis 10. **Tolerance**, as defined by either of the following: a. A need for markedly increased amounts of cannabis to achieve intoxication or desired effect b. Markedly diminished effect with continued use of the same amount of cannabis 11. **Withdrawal**, as manifested by either of the following: a. At least 3 of the following signs and symptoms develop within approximately 1 week after cessation of cannabis use that has been heavy and prolonged (i.e.—usually daily or almost daily use over a period of at least a few months): i. Irritability, anger, or aggression ii. Nervousness or anxiety iii. Sleep difficulty (e.g.—insomnia, disturbing dreams) iv. Decreased appetite or weight loss v. Restlessness vi. Depressed mood vii. At least 1 of the following physical symptoms causing significant discomfort: a) Abdominal pain b) Shakiness/tremors c) Sweating d) Fever e) Chills f) Headaches b. Cannabis (or a closely related substance) is taken to relieve or avoid withdrawal symptoms
**Severity specifiers**
Specify current severity based on the following guidelines: 1. Mild: Presence of 2 or 3 symptoms 2. Moderate: Presence of 4 or 5 symptoms 3. Severe: Presence of 6 or more symptoms

## Cannabis and Eating Behaviors

THC use has been commonly reported to contribute to increased appetite, overconsumption, and heightened appreciation of food– what is colloquially referred to as “the munchies” [18]. Unfortunately, most of the literature about the role of cannabis in appetite and eating behaviors has been based on research in animal models, which may be somewhat limited in generalizability to humans due to interspecies differences in reward-based eating, the role of taste in food selection, and ability to perceive different flavors [19]. Further, the limited research on this topic with human participants is quite dated. Noting this gap in the literature, Roberts and colleagues [18] studied the subjective experience of “the munchies” in frequent cannabis users with a questionnaire about their perceived eating behavior while under the influence of cannabis. They found that cannabis use was associated both with enhancing the motivational factors influencing the initiation of eating, and hedonic factors involved in maintaining eating. Motivational factors included increased hunger, salience of food, and perceived capacity for food (e.g., eating beyond fullness) [18]. Hedonic factors included enhanced smell, taste, and appreciation of food [18]. Despite findings highlighting cannabis’ acute effects on multiple factors influencing eating behaviors, there was no correlation between BMI and scores on a questionnaire measuring these motivational and hedonic factors [18].

Another recent study by Weltens and colleagues provided preliminary mechanistic evidence for the role of THC in “the munchies” among individuals within the normal BMI range who were not cannabis users [19]. Participants (N = 17) had four test sessions in which they were either given THC orally, THC via intragastric infusion, placebo orally, or placebo via intragastric infusion. In each test session, they rated their levels of “liking” and “wanting” in response to images depicting foods in various calorie ranges, and later, they consumed as much of a milkshake as they desired until satiated. Authors found that THC was associated with increased “liking” and “wanting” high calorie foods, and THC administered orally (vs. intragastrically) increased the amount of milkshake consumed. These findings provide support for the role of THC in increasing anticipatory food reward responses, and in increasing high calorie food consumption specifically when consumed orally. While this was a small trial, it appears that orosensory stimulation was an important factor increasing food intake among those who had been administered THC [19]. These results are consistent with the one known animal study that parsed out the orosensory stimulation of feeding using a sham protocol [20]. This study demonstrated that the presence of fat in rats’ mouths induced cephalic phase responses (i.e., salivation, gastric acid secretion, insulin release, etc.) that stimulated the intestinal endocannabinoid system and in turn, increased food consumption [20]. Thus, Welten and colleagues’ [19] study extends these findings and suggests that the orosensory stimulation of edible cannabis may influence food consumption in humans as well.

Consistent with the results of the aforementioned study, another study found correlations between cannabis use and various dietary patterns. Specifically, at baseline, participants who were more frequently using edible cannabis reported more frequent consumption of higher fat food and greater calorie intake [21]. When dietary patterns were then tracked using daily food diaries over the course of a month, cannabis users also had higher intake of salty snacks and fast food compared to cannabis non-users, although this difference was small [21]. Despite these differences in eating behaviors, BMI was not significantly different between users and non-users, which the authors speculated could be due to both groups being physically active [21].

## Cannabis and Eating Disorders

Multiple studies have reported on cannabis use among individuals with eating disorders (EDs), particularly binge eating disorder (BED). Binge eating disorder is defined as at least weekly episodes of consuming unusually large amounts of food in a short period of time with a feeling of loss of control (LOC) over food intake, and at least three associated symptoms (e.g., eating more rapidly than usual, eating until uncomfortably full) [17].

In a study of adults with at least weekly binge eating, cannabis use frequency did not predict eating disorder symptoms [22]. Of note, in this study, cannabis use was measured with a questionnaire yielding scores that reflected level of risk (i.e., low, moderate, high) for CUD, and no participants were deemed high risk for CUD based on their responses [22].

In a study of college students, cannabis use did not predict eating pathology, but problematic cannabis use (i.e., negative consequences such as lower energy, driving while intoxicated) was associated with eating pathology, via LOC eating [23]. In another study of college students who engaged in heavy alcohol use, those who screened positive for an ED (including anorexia nervosa [AN], bulimia nervosa [BN], and binge eating behaviors) reported significantly greater frequency of cannabis use and greater CUD symptom severity compared to those who did not screen positive for EDs [24]. In an examination of individual ED screening responses, authors also found that those screening positive for CUD were more likely to report LOC eating. There was a marked difference between results for men and women in this study, such that 50% of men with positive ED screens met criteria for CUD compared to 16% of women [24]. These results suggest that while EDs are less common in men, there appears to be a high overlap between EDs and CUD in this population.

In a systematic review and meta-analysis, CUD was one of the most common co-morbid diagnoses in individuals meeting criteria for an ED including AN, BN, BED, and eating disorder not otherwise specified (EDNOS) [25]. Specifically, across 9 studies examining current prevalence, 20.9% of those with EDs had CUD over the past 12 months [25]. Those with binging and purging behaviors were more likely to evidence substance use disorders including CUD, which the authors postulated was due to this population having greater difficulty with emotion regulation and impulse control [25]. Authors explained that symptom substitution can occur in ED recovery, such that when individuals are no longer binging and purging to cope with negative emotional states, they may turn to another maladaptive coping strategy such as substance use [25]. Another potential explanation for the overlap between CUD and EDs is common underlying difficulties with altered reward sensitivity, such as heightened focus on the rewarding aspects of food or cannabis coupled with lower concern about potential problematic consequences [24]. When negative emotions such as guilt or depression follow either problematic eating or cannabis use, these behaviors may then be used for emotion regulation, thus creating a negative feedback loop [24].

## Cannabis and Physical Activity

Another factor to consider when examining the role of cannabis in weight is the extent to which it may influence physical activity. There have been anecdotal reports of cannabis decreasing motivation for physical activity, particularly when cannabis strains with a more calming effect are used (e.g., *Cannabis indica*) [26].

A recent systematic review of 97 studies (with sample sizes ranging from 88–653,211) found that among adolescents, cannabis use was negatively associated with physical activity and positively associated with sedentary behavior [27]. In a large population-based study of adults (N = 12,618), current and past cannabis users had a lower prevalence of self-reported moderate or vigorous physical activity compared to non-users [28]. Additionally, as frequency of cannabis use increased, time spent engaging in physical activity decreased [28]. However, in another smaller study (N = 98), there was no difference in exercise behavior (e.g., total number of days and minutes of moderate to vigorous exercise) between cannabis users and non-users [21]. Given these conflicting findings, additional research about the role of cannabis use in physical activity is needed.

## Cannabis and Weight

In light of aforementioned research suggesting a relationship between cannabis use, eating behaviors, eating disorders, and physical activity, it would logically follow that cannabis use would be associated with weight gain and obesity. However, recent research has not supported this notion. Building upon older cross-sectional research which found a lower prevalence of obesity in cannabis users compared to non-users [29, 30], Alshaarawy and Anthony [31] conducted a large prospective study dawn from the National Epidemiologic Survey on Alcohol and Related Conditions. In this study, there were two waves of data collection three years apart. At both timepoints, cannabis use and BMI were collected. Cannabis use was categorized as: 1) lifetime never use, 2) discontinuation/quitting (used at the first time point but not in the 12 months preceding the second timepoint), 3) initiation (no use at the first time point, but did use in the 12 months preceding the second timepoint), and 4) persistent use (used at both timepoints). Consistent with previous epidemiological studies, they found that BMI increased over time among all groups, but that all cannabis subgroups had an attenuated BMI gain compared to never users [31]. Authors speculated that this counterintuitive finding could be due to downregulation and reduced expression of CB1 in persistent cannabis users, and/or due to the anti-inflammatory effects of CB2. Indeed, the anti-obesity medication Rimonabant, which was approved in Europe but then taken off the market several years ago due to adverse psychological events, mimicked THC by chronically stimulating CB1 receptors, resulting in reduced energy storage, increased metabolic rates, and ~ 10% weight loss [32].

In another large prospective study with a 30-year follow-up, Jakob and colleagues [33] examined the associations between BMI and cumulative cannabis exposure from early adulthood through middle age. Current cannabis use was defined as use over the past 30 days at each follow-up timepoint (baseline and years 2, 5, 7, 10, 15, 20, 25, and 30). Cumulative cannabis exposure was defined as the total number of days of cannabis use, which was calculated based on the average reported frequency of cannabis use in the month prior to each timepoint. Consistent with Alshaarawy and Anthony’s [31] findings, Jakob and colleagues found that current cannabis use was associated with lower BMI. Novel findings were that 1) cumulative cannabis use is not associated with lower BMI, and 2) the BMIs of individuals who quit using cannabis did not increase. This latter result was contrasted with other research finding an association between tobacco smoking and lower BMI. That is, current tobacco use is associated with lower BMI, but when tobacco users quit, BMI increases [33]. If cannabis use caused lower weight, it would follow that BMI would similarly increase with cannabis cessation. Because this study did not find such a pattern, Jakob and colleagues [33] concluded that the link between cannabis use and BMI is unlikely to be causal, but rather due to residual confounding. There are many potential confounding variables that make cannabis users versus never users different, and these factors cannot all be accounted for in observational studies. They further stated, “Recreational cannabis use may not result in clinically relevant changes in BMI and that the association between current cannabis use and lower BMI can likely be explained by residual confounding… This should reassure health authorities, medical professionals, and the public that increases in cannabis use in the general population might not lead to higher BMI, but also dampen enthusiasm that cannabis use might be solution to the obesity epidemic” [33, p.46].

Although most research relies upon observational studies, which are prone to bias and cannot determine causality, one recent study used the Mendelian randomization method in which genetic variants are examined for any potential causal association, in a manner analogous to a randomized controlled trial [34]. Alayash and colleagues [34] found that there was no evidence to support a causal relationship between lifetime cannabis use and BMI, waist circumference, or waist-to-hip ratio. Of note, “lifetime cannabis use” was defined broadly in this study, and a distinction was not made between THC vs. CBD.

Studies that do tease apart THC and CBD are particularly valuable. A systematic review by Pinto and Martel [8] examined 11 trials about the influence of CBD on appetite and body weight. Of these, the majority of studies found that CBD was associated with decreased appetite and/or increased fullness. There was only one study that found CBD to be associated with increased appetite, and two studies did not find any association with appetite [8]. Only three studies reported on body weight, and while there were generally no significant differences found between appetite and body weight, one study [35] found that participants with higher baseline BMI had greater weight loss following CBD treatment.

Overall, literature on the role of cannabis in weight, particularly in human studies, is nascent and challenged by several limitations, such as the presence of confounding variables. For example, cannabis users frequently smoke cigarettes and/or smoke cannabis mixed with tobacco [36], and as noted previously, tobacco has been known to decrease body weight [34, 37]. Additionally, particularly in large epidemiological studies, cannabis use is assessed broadly (e.g., “never” vs “lifetime use”), without distinctions made regarding frequency of use, route of administration, cannabinoid content, whether CUD is present, and other relevant variables. Cannabinoid content is a particularly important variable given that, as previously noted, THC and CBD have different and perhaps opposite effects on appetite. Furthermore, given ED research suggesting a positive correlation between CUD and LOC eating, CUD is an important diagnosis to consider in future research on weight and weight-related behaviors.

## Clinical Considerations

While more nuanced research is needed to guide clinical recommendations, the state of the current literature does suggest some key areas for screening and patient education (see Table 2 for a visual representation of recent findings). When treating individuals presenting for weight management concerns, assessing for use of substances, including cannabis, is considered standard practice. However, this review suggests that asking broadly about whether patients use cannabis is not enough. Assessing for problematic cannabis use, including CUD, has the greatest clinical relevance as it relates to eating disorders. For those meeting criteria for CUD, targeted substance abuse treatment will be an important first step in ED recovery, and transferrable skills gained from substance abuse treatment may then assist those with EDs in improving emotion regulation without substances or food.

**Table 2:** Recent studies examining the relationship between cannabis, weight, and weight-related behaviors

	Cannabis Use				References		
	“Cannabis” use defined broadly	THC	CBD	Problematic Cannabis Use/Cannabis Use Disorder		Strengths	Limitations
Appetite/Cravings			⬇		[8] *	• Systematic review • Specified THC vs CBD	• In the 11 studies included, 5 different dosages of CBD were used
		⬆			[18]	• Only recent study to explore subjective experience of “the munchies” in humans	• Relied on retrospective report of experiences when using cannabis
		⬆			[19]	• Randomized placebo trial • Distinguished between oral and intragastric THC administration	• Small study (N = 17)
Overconsumption		⬆			[18]	-	-
		⬆			[19]	-	-
Unhealthy Food Choices	⬆	⬆			[21]	• Specified THC vs CBD • Used daily food diaries	• Lack of participant blinding • No standardized dosing procedure • Baseline physical activity level was higher than standard guidelines
Physical Activity	⬇				[27]*	• Systematic review	• All studies were observational • Evidence was not graded • Effect sizes were not estimated
	⬇				[28]	• Large, nationally representative, population-based sample	• Based on self-report
	═				[21]	-	-
Eating Disorders	═				[22]	• Used a clinical binge eating sample	• Included small sample of individuals using cannabis, and none were deemed high risk for cannabis use disorder
				⬆	[23]	• Explored loss of control eating as a mediator	• College student sample • Cross-sectional design
				⬆	[24]	• Sample had a higher percentage of men than typically captured in eating disorders research, which allowed for examination of sex differences	• Sample of college students who were also drinking alcohol heavily
				⬆	[25]*	• Systematic review and meta-analysis using PRISMA	• Over-representation of young female participants in studies reviewed
Weight			═/⬇		[8]	-	-
	═				[18]	-	-
	═				[21]	-	-
	⬇				[31]	• Large national sample • Prospective design	• Cannabis use and BMI based only on self-report
	═/⬇				[33]	• Large prospective study • 30-year follow up	• Based on self-report, w/potential for underreporting particularly where cannabis is/was previously illegal over the past 30 years
	═				[34]	• Large sample • Mendelian randomization approach used to examine possible causal associations	• Based on self-report • Did not gather information about dose, frequency, or duration of use to determine if there is a dose-dependent relationship

* = Systematic review.- = strengths/limitations previously noted in the table. ⬆ = positive correlation. ⬇ = Negative correlation. ═ = No significant association found

This review also suggests that it is critical to inquire about the specific cannabinoids used. THC is associated with increased motivation for food, reward-based eating, and overconsumption. On the other hand, CBD may have less of an influence on appetite, or even suppress appetite, as well as reduce inflammation. Thus, while the state of the research is not strong enough for practitioners to specifically recommend CBD for weight management, patients who desire to lose weight and do plan to continue to use cannabis should be counseled about the relatively higher risk of THC on increased appetite, LOC eating, and unhealthy eating behaviors, as compared to CBD.

Furthermore, route of administration appears to make a difference. Whereas inhaled cannabis leads to rapid onset of increased appetite over a shorter duration, edible cannabis leads to delayed but stronger effects on appetite. On the other hand, edible cannabis may contain sugar and other ingredients that are counterproductive to weight loss goals, and inhaled cannabis is associated with respiratory risks [38]. All of these factors considered, practitioners are left without an empirically-based recommendation about the safest route of administration, but should educate patients about the risks of all routes.

When educating patients about the influence of cannabis on weight, it would be accurate to concede that the literature is mixed. Some human studies point to an association between cannabis use and lower BMI, but these are limited by confounding variables and lack of nuance regarding the important factors (e.g., frequency and duration of use, cannabinoid profile, route of administration) previously discussed. Still, research does show that consistent with anecdotal reports, cannabis seems to drive consumption of less healthful options including foods higher in fat, salt, and calories. While research is mixed, cannabis use also appears associated with reduced physical activity. Therefore, weight may not be the most important outcome to consider. The impact of cannabis on engagement in healthy behaviors, and the downstream effects on cardiometabolic functioning, are also worth exploring with patients as they make informed decisions about whether cannabis fits with their health goals. Future research should explore how cannabis use is associated with other health indicators beyond BMI.

## Conclusion

In sum, the state of the literature regarding the relationship between cannabis, weight, and weight-related behaviors is complex. Most studies point to a relationship between cannabis use and lower BMI, although causality is difficult to determine due to the presence of confounding variables in observational studies. Perhaps of even greater concern, the vast majority of studies do not differentiate between cannabinoid profile or route of administration, which is a major limitation considering that THC vs CBD, and inhaled vs edible cannabis appear to have very different influences on appetite. Furthermore, those with problematic cannabis use or meeting criteria for CUD are at higher risk for disordered eating, such as LOC eating. As cannabis continues to increase in legality, acceptability, and prevalence amidst an obesity epidemic, additional research is needed to better understand the specific factors (e.g., frequency of use, cannabinoid profile, route of administration, CUD) that influence weight and weight-related behaviors.

## Key References

- Gibson LP, Skrzynski CJ, Giordano GR, Bryan AD. A daily diary investigation of cannabis use and its diet and exercise correlates. Front Psychol. 2023 Aug 4;14:1217144. doi:10.3389/fpsyg.2023.1217144.
  - This research highlights the relationship between cannabis use and unhealthy food choices.
- Pinto JS, Martel F. Effects of cannabidiol on appetite and body weight: A systematic review. Clin Drug Investig 2022: 42(11):909–919. doi: 10.1007/s40261-022–01205-y.
  - Unlike most other cannabis research that does not differentiate between cannabinoids, this systematic review specifically examined the relationship between CBD, appetite, and body weight.
- Jakob J, Schwerdtel F, Sidney S, Rodondi N, Pletcher MJ, Reis JP, Muniyappa R, Clair C, Tal K, Bancks MP, Rana JS, Collet T-H, Auer R. Associations of cannabis use and body mass index-The Coronary Artery Risk Development in Young Adults (CARDIA) study. Eur J Intern Med 2024:129:41–47. doi: 10.1016/j.ejim.2024.07.007.
  - In this 30-year prospective study, current cannabis use was found to be associated with lower BMI, but cumulative cannabis use was not. Additionally, cannabis cessation did not result in increased BMI. Results suggest the link between cannabis use and BMI is unlikely to be causal.
- Roberts CA, Jager G, Christiansen P, Kirkam TC. Exploring the munchies: An online survey of users’ experiences of cannabis effects on appetite and the development of a Cannabinoid Eating Experience Questionnaire. J Psychopharmacol 2019:33(9):1149-1159. doi: 10.1177/0269881119862526.
  - This study explores the subjective experience of “the munchies,” including factors that influence the initiation and maintenance of eating.

## Data Availability

No datasets were generated or analysed during the current study.
